# Comparison of the efficacy of laser-activated and ultrasonic-activated techniques for the removal of tricalcium silicate-based sealers and gutta-percha in root canal retreatment: a microtomography and scanning electron microscopy study

**DOI:** 10.1186/s12903-021-01638-5

**Published:** 2021-05-22

**Authors:** Ruiqi Yang, Yuqing Han, Zhaohui Liu, Zhezhen Xu, Hongyan Liu, Xi Wei

**Affiliations:** 1 Department of Operative Dentistry and Endodontics, Guanghua School of Stomatology, Hospital of Stomatology, Sun Yat-sen University, Guangzhou, Guangdong Province China; 2 Guangdong Provincial Key Laboratory of Stomatology, Guangzhou, Guangdong Province China

**Keywords:** Passive ultrasonic irrigation, Photon-initiated photoacoustic streaming, Endodontic retreatment, iRoot SP, Micro-CT, Scanning electron microscopy

## Abstract

Tricalcium silicate-based sealers have been usually indicated for the single-cone technique and result in more residual filling materials in root canal retreatment. Passive ultrasonic irrigation and photon-initiated photoacoustic streaming have been reported to improve the removal efficacy of root canal filling materials. However, the abilities of both techniques combined with NiTi re-instrumentation to remove residual tricalcium silicate-based sealer and gutta-percha have not been compared. The aim of this study was to evaluate the efficacy of laser-activated and ultrasonic-activated techniques in vitro for the removal of the tricalcium silicate-based sealer iRoot SP and gutta-percha after standard canal retreatment procedures with the use of nickel-titanium (NiTi) rotary instruments.

## Methods

Thirty-six extracted single-rooted teeth were filled using a single-cone technique with GP and iRoot SP sealer. These root canals were then retreated using the ProTaper Universal retreatment (PTUR) system. The samples were divided into three groups according to the final irrigation techniques used in retreatment procedures: group 1, classic syringe-based irrigation (CSI); group 2, passive ultrasonic irrigation (PUI); and group 3, photon-initiated photoacoustic streaming (PIPS). All groups were irrigated with 2.5% sodium hypochlorite and 17% EDTA solutions. Micro-CT scans were taken to evaluate the volume of root filling materials. The cleanliness of root canal walls was scored by scanning electron microscopy (SEM).

## Results

All groups had residual root filling materials in the root canals after mechanical retreatment. Additional use of PIPS removed significantly higher volume of root fillings than PUI and CSI techniques (*p* < 0.05). SEM scores were significantly lower in the PIPS group than in the PUI and CSI groups, especially in the middle and apical thirds (*p* < 0.05).

## Conclusions

None of the additional techniques in this study completely removed the residual iRoot SP and gutta-percha. Compared to PUI and CSI, activation of 2.5% sodium hypochlorite and 17% EDTA with PIPS greatly improved the removal of the residual iRoot SP and gutta-percha following NiTi mechanical retreatment.

## Background

Nonsurgical root canal retreatment is an important treatment for persistent periapical periodontitis. The procedures involve reaccessing the root canal system, complete removal of root filling materials, disinfection and root canal refilling to allow periradicular healing [1–3]. Filling materials remaining on the root canal walls can harbor microorganisms and lead to retreatment failure. Removal of the existing root filling materials is therefore key for the long-term success of root canal retreatment, and can be affected by the type of retreatment technique. Several techniques have been used for removing root filling materials including solvents, hand instruments, ultrasonic instruments and nickel–titanium (NiTi) rotary systems [4–6]. The use of NiTi rotary files such as ProTaper Universal retreatment (PTUR) rotary files, is favored over the use of traditional hand instruments due to the lower amount of time required for retreatment procedures [7]. Passive ultrasonic irrigation (PUI) is an ultrasonic-activated treatment modality for removing bacteria, smear layers and dental debris [8, 9] through acoustic streaming and cavitation with noncutting action to irrigation solution in the root canal. Some researchers have reported that ultrasonic irrigation for retreatment had a superior effect on removing the sealer and smear layer after post space preparation [10–12]. PUI used after NiTi rotary instruments enhanced the removal of the filling materials more than the use of Reciproc R25 or TS2 alone [13]. However, neither PUI nor NiTi instrumentation can completely remove the gutta-percha and sealers from root canals [14].

iRoot SP (Innovative Bioceramix, Vancouver, BC, Canada), also known as EndoSequence BC Sealer (BC Sealer, Brasseler USA, Savannah, GA, USA), is a novel tricalcium silicate-based sealer with favorable biocompatibility and antibacterial properties. Because high temperature can influence the setting time, flow and porosity of iRoot SP, it is usually indicated for the single-cone technique [15]. Canals obstructed with the single-cone technique always contain a larger amount of sealer than those treated with other filling techniques, such as lateral and warm vertical techniques [16]. Some researchers have demonstrated that BC sealer resulted in more residual filling materials than AH Plus in root canal retreatment [17]. Simsek et al. found that iRoot SP and/or gutta-percha could not be completely removed by R-endo and ultrasonic irrigation and that canals in all groups tended to amass more debris in the apical third. Therefore, a more effective technique should be developed in future investigations [18].

Photon-initiated photoacoustic streaming (PIPS) is a new laser-activated technique that is used with a low-energy erbium: yttrium-aluminum-garnet (Er:YAG) laser to activate the irrigant in the root canal [19]. PIPS allows for deeper penetration in dentinal tubules of irrigant and can disinfect the dentinal tubules [20, 21]. Studies have reported that PIPS can remove the smear layer and debris more effectively than syringe-based irrigation and ultrasonic activation [22, 23]. PIPS uses only a laser fiber tip placed inside the access cavity, avoiding the risk of thermal damage of the teeth and periodontal tissue. The combined use of PIPS with a NiTi rotary system to remove AH Plus, MTA Fillapex and EndoSequence BC is more effective than the use of NiTi alone [24]. PIPS performs better than ultrasonic techniques in removing AH Plus sealer from oval root canals [25]. However, to the best of our knowledge, the abilities of laser and ultrasonic-activated techniques combined with NiTi re-instrumentation to remove residual tricalcium silicate-based sealer and gutta-percha have not been compared.

This study was aimed to assess the effectiveness of PIPS and PUI for the removal of iRoot SP sealer and gutta-percha after mechanical retreatment. Classic syringe-based irrigation(CSI) was used as a negative control. Microcomputed tomography (micro-CT) and scanning electron microscopy (SEM)-based quantification of residual filling materials were used to evaluate the removal of filling materials and the cleanliness of the root canal walls. The null hypothesis tested was that there was no significant difference among the three retreatment techniques.

## Methods

### Sample size calculation

Based on the data of a previous study [26], the sample size in the present study was calculated by the PASS 15 software (Power Analysis & Sample Size, NCSS, USA). In the ANOVA study, sample size of 12, 12 and 12 were obtained from the 3 groups. The total sample of 36 subjects achieves 86% power to detect differences with a 0.0500 significance level.

## Sample selection

The study was approved by the local ethics committee (KQEC-2020-07) and written informed consent was obtained from all patients. Thirty-six extracted human teeth with completely developed apices and a single straight root canal were selected for the study. Micro-CT (Scanco Medical, Zurich, Switzerland) scan was performed to verify a single root canal with a curvature less than 15° angle and a ratio of the buccolingual to mesiodistal dimensions of less than 2:1 at 5 mm from the root apex for each tooth [27, 28]. Teeth with previous root canal treatment, calcification, and resorption were excluded. Specimens were stored in a 0.5% chloramine-T solution at 4 °C until use.

## Root canal preparation

Straight access cavities were prepared with a diamond fissure bur SF-41(MANI, INC., Japan) under cooling with water spray. All the teeth were decoronated with the same bur, and the root was cut to a 13 mm length. Working length (WL) was determined by subtracting 1 mm from the length at which a size #10 K-file first appeared at the apical foramen.

The root canals were prepared using ProTaper Next (PTN) rotary instruments (Dentsply, Maillefer, Ballaigues, Switzerland, Switzerland) up to X3 (#30/0.07) to the WL [24], driven by a torque-controlled motor (SybronEndo, CA, U.S.A.) at 300 rpm with the crown-down technique. The root canals were irrigated by 2 mL of 2.5% sodium hypochlorite (NaOCl) (Disineer, Shandong, China) with a 30-gauge side-vented needle (United Dental, Shanghai, China) between each instrument. The final irrigation was 2 mL 17% ethylenediaminetetraacetic acid (EDTA) (Longly, Wuhan, China) for 1 min, followed by 2 mL of 2.5% NaOCl for 1 min and a rinse with 2 mL of saline solution for 1 min[24, 29]. Then, the root canals were dried with sterile paper points (Dayading, Beijing, China).

## Root canal filling

The canals were obturated using single-cone technique with tricalcium silicate-based sealer-iRoot SP and gutta-percha. The sealer was injected into the root canal with a plastic tip, and the tip was slowly pulled toward the orifice from the middle third in the canal. Then, a master cone (Dentsply, Maillefer, Ballaigues, Switzerland, Switzerland) was inserted into the root canal to the WL and was cut at the orifice level using a heated plugger. The coronal openings were sealed with Caviton (GC, Tokyo, Japan). All samples were then stored at 37 °C and 100% relative humidity for 2 weeks to allow complete sealer setting. To avoid the inter-operator variability, the same skilled operator performed all root canal filling.

## Root canal retreatment

### ProTaper Universal retreatment (PTUR) rotary instrumentation

Root canal retreatment was performed using ProTaper Universal retreatment (PTUR) rotary files (Dentsply, Maillefer, Baillaigues, Switzerland) according to the manufacturer’s instructions. The D1 (#30/0.09), D2 (#25/0.08) and D3 (#20/0.07) files were sequentially used for the coronal, middle and apical thirds with the crown-down technique, respectively, and no solvent was used. The root canal was further prepared with PTN X3 (#30/0.07) and X4 (#40/0.06) to the WL. Each set of instruments was used for 6 canals. Then, all samples were irrigated with 2 mL of 17% EDTA for 1 min, 2 mL of 2.5% NaOCl for 1 min, and 2 mL of saline solution for 1 min. Irrigation was performed with syringes and 30-gauge side-vented needles. The samples were dried with sterile paper points.

## Final irrigation technique

After instrumentation, the samples were divided into 3 groups according to the random number table method (n = 12):

Group 1: classic syringe-based irrigation (CSI).

Group 2: passive ultrasonic irrigation (PUI).

Group 3: photon-initiated photoacoustic streaming (PIPS).

**Group 1**-Classic syringe-based irrigation (CSI).

The root canals were irrigated with 3 mL of 2.5% NaOCl and 3 mL of 17% EDTA, for 40 s respectively with a 30-gauge side-vented needle. The tip was placed 1 mm short of the WL and moved up and down within 4 mm in the root canal. This group was defined as the negative control.

**Group 2-**Passive ultrasonic irrigation (PUI).

The root canals were irrigated in 5 s irrigation and then 5 s activation with 4 times repetitions with 3 mL of 2.5% NaOCl. The activation was performed by using a K-type noncutting ultrasonic size 15 tip (Satelec Acteon, Mérignac, France) at 30%-unit power. The ultrasonic tip was placed 1 mm short of the WL without touching the root canal walls. After that, 3 mL of 17% EDTA was introduced into the root canals using the same procedure. The contact time of each solution with dentin surfaces was standardized at 40 s.

**Group 3-**Photon-initiated photoacoustic streaming (PIPS).

The root canals were sequentially rinsed with 3 mL of 17% EDTA and 3 mL of 2.5% NaOCl and were activated by an Er:YAG laser (Fotona, Ljubljana, Slovenia) with a 300-µm endodontic fiber tip (20 mJ, 15 Hz, 50-µs pulse, average power, 0.3 W). The tip was placed in the access cavity. The irrigation and activation process were performed using the same protocols in Group 2. Finally, the root canals of the three groups were irrigated with 3 mL of distilled water and dried with sterile paper points. A single operator prepared all specimens.

## Micro-CT analysis

The specimens were scanned with a Scanco µCT50 micro-CT (Scanco Medical, Brüttisellen, Switzerland) four times during the treatment: before instrumentation, after the root canal filling, after mechanical retreatment and after the final irrigation procedures. Silicone moulds were created and served as a sample container that allowed for scanning teeth in the same position during the scanning procedure. All samples were scanned at the same position and radiation settings with a voxel size of 34.4 μm, 250 projections, 70 kV, and 57 mA. The volume of the filling materials was measured with Mimics Research 20.0 software. The removed volume for the filling materials used in the final irrigation procedures was calculated by subtracting the volume of the remaining filling materials after the final irradiation procedures from the volume of the remaining filling materials after the mechanical retreatment.

## Scanning electron microscopy evaluation

After micro-CT scanning, scanning electron microscopy (SEM) (Hitachi, Tokyo, Japan) was used to evaluate the cleanliness level of the root canal walls. A shallow longitudinal groove along the buccolingual direction was made in each specimen using a diamond disc (MANI, INC., Japan). A chisel was used to split the teeth into two halves longitudinally. All samples were dehydrated in a desiccator for 24 h and then sputter-coated with gold (Hitachi, Tokyo, Japan). Then, the coronal, middle and apical thirds of all samples were observed by SEM at 10 kV with a magnification of 1000×. Scanning electron micrographs of at least three randomly selected areas from each sample were taken. The SEM images were scored blindly by two endodontist using modified criteria based on Bernardes et al. and Pirani et al. as follows [4, 30]: 0, absence of smear layer and filling debris, more than 75% of the tubules exposed and opened; 1, smear layer and filling debris present in limited areas, < 75% of tubules exposed; 2, smear layer and filling debris often present, < 50% of tubules visibly exposed in a limited area; and 3, smear layer and filling debris present above all dentin, no tubules visible.

### Statistical analysis

The normality and the equality of the data’s variance were evaluated using the Shapiro–Wilk test and Levene’s test. The Kruskal-Wallis H test and Mann-Whitney U test were used to evaluate statistically significant differences of volume of the residual filling materials, volume of the material removed by final irrigation and SEM scores in three parts of the canals among the three groups, and a Friedman and pairwise signed-rank tests were used to compare the difference of root third in each group. The Wilcoxon signed-rank test was used to compare the differences of the volume of remaining filling materials in the coronal, middle and apical thirds before and after the final irrigation-activation technique within each retreatment group. A value of *p* < 0.05 was considered statistically significant. The data were analyzed by IBM SPSS Statistics 20.0 (IBM SPSS Inc., Chicago, IL, USA).

## Results

### Micro-CT imaging and analysis of the filling materials

**Fig. 1:**
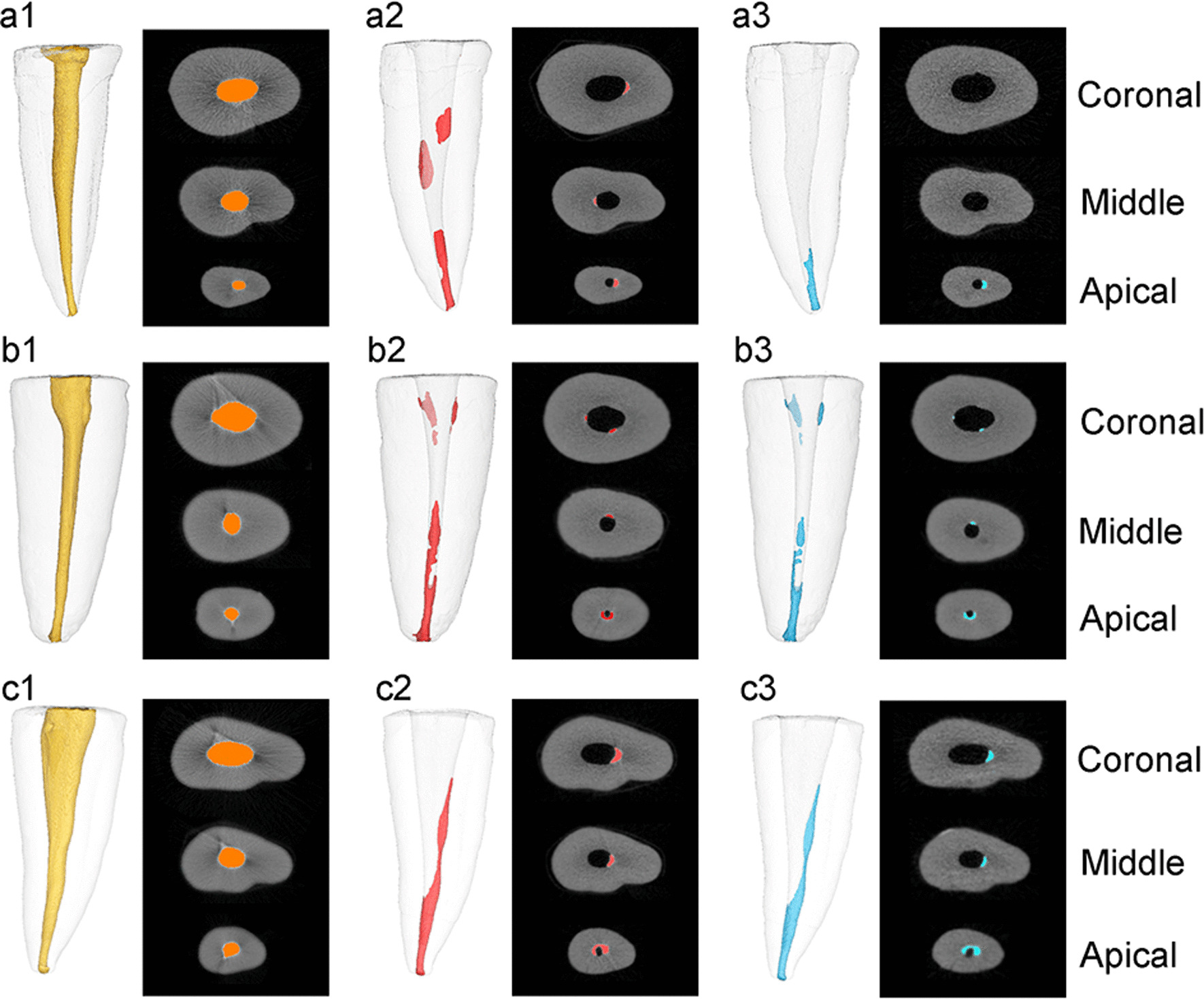
Three-dimensional imaging of micro-CT scans of the remaining filling materials: **a** photon-induced photoacoustic streaming (PIPS) group (**a1** after obturation; **a2** after mechanical retreatment; **a3** after irrigation); **b** passive ultrasonic rinsing (PUI) group (**b1** after obturation; **b2** after mechanical retreatment; **b3** after irrigation); **c** classic syringe-based irrigation (CSI) group (**c1** after obturation; **c2** after mechanical retreatment; **c3** after irrigation)

All specimens had residual filling materials after all treatment procedures (Fig. 1). Median, minimum and maximum values of the volume of the remaining filling materials after mechanical retreatment and after the final irrigation technique are shown in Table 1. There was no statistically significant difference in the remaining filling materials overall or for each third of the root canal after mechanical retreatment among the three groups (*p* > 0.05). Similar results were observed after final irrigation among the three groups (*p* > 0.05). However, the remaining filling materials after final irrigation in the PUI and PIPS groups were significantly less than those after mechanical retreatment (*p* < 0.05). For the CSI group, there were significantly less residual filling materials in the middle and apical parts than after mechanical retreatment (*p* < 0.05), whereas no significant differences were detected in the coronal third (*p* > 0.05).

**Table 1:** Volumes of residual filling materials (mm^3^) measured by micro-CT analysis in each group

Groups	CSI	PUI	PIPS
	Median (Minimum–Maximum)		
*After mechanical instrumentation*			
Coronal	0.02 (0.00–0.30)^a^	0.04 (0.00–0.64)^a^	0.10 (0.01–0.46)^a^
Middle	0.21 (0.00–0.56)^a^	0.10 (0.00–0.60)^a^	0.29 (0.01–0.99)^a^
Apical	0.34 (0.04–0.63)^a^	0.49 (0.00–0.66)^a^	0.30 (0.11–0.75)^a^
Overall	0.63 (0.12–1.15)^a^	0.68 (0.09–1.55)^a^	0.75 (0.39–1.84)^a^
*After final irrigation*			
Coronal	0.01 (0.00–0.30)^a^	0.01 (0.00–0.62)^b^	0.01 (0.00–0.25)^b^
Middle	0.14 (0.00–0.38)^b^	0.04 (0.00–0.22)^b^	0.06 (0.00–0.80)^b^
Apical	0.33 (0.04–0.62)^b^	0.39 (0.00–0.65)^b^	0.23 (0.06–0.67)^b^
Overall	0.52 (0.08–1.15)^b^	0.50 (0.03–1.19)^b^	0.44 (0.20–1.43)^b^

ab Ranking: statistically significant differences among after mechanical retreatment and after final irrigation in the CSI, PUI and PIPS groups (*p* < 0.05)

The filling materials volume removed by the final irrigation in the three groups is shown in Table 2. Significant differences were observed when comparing the volumes of filling materials removed by the final irrigation technique among the three groups (*p* < 0.05), with the PIPS group showing the most and the CSI group showing the least in the overall canal. PIPS could remove more residual filling materials than CSI and PUI (both *p* < 0.05) in the coronal third. In the middle and apical thirds, PIPS also removed significantly more residual filling materials than CSI (*p* < 0.05), whereas the difference between the PIPS and PUI groups were not significant (both *p* > 0.05). Significantly more material was removed in the PUI group than the CSI group in the apical third (*p* < 0.05). In terms of different parts of the root canal under the same final irrigation technique, PIPS and CSI equally removed the residual iRoot SP and gutta-percha in different third of the root canal (*p* > 0.05). PUI removed more residual material in the middle and apical thirds, than in the coronal third (*p* < 0.05).

**Table 2:** Volumes of residual filling materials (mm^3^) removed by the final technique measured by micro-CT analysis in each group

Groups	CSI	PUI	PIPS
	Median (Minimum–Maximum)		
Coronal	0.03 (0.00–0.09)^a1^	0.02 (0.00–0.05)^a1^	0.10 (0.00–0.29)^a2^
Middle	0.01 (0.00–0.02)^a1^	0.10 (0.00–0.59)^b12^	0.14 (0.00–0.32)^a2^
Apical	0.01 (0.00–0.02)^a1^	0.07 (0.00–0.19)^b2^	0.06 (0.00–0.13)^a2^
Overall	0.03 (0.00–0.67)^1^	0.14 (0.00–0.32)^2^	0.30 (0.09–0.50)^3^

ab Ranking: statistically significant differences among the coronal, middle, and apical thirds within the CSI, PUI and PIPS groups (*p* < 0.05)

123 Ranking: statistically significant differences among the PIPS, PUI and CSI groups (*p* < 0.05)

## SEM imaging and evaluation

**Fig. 2:**
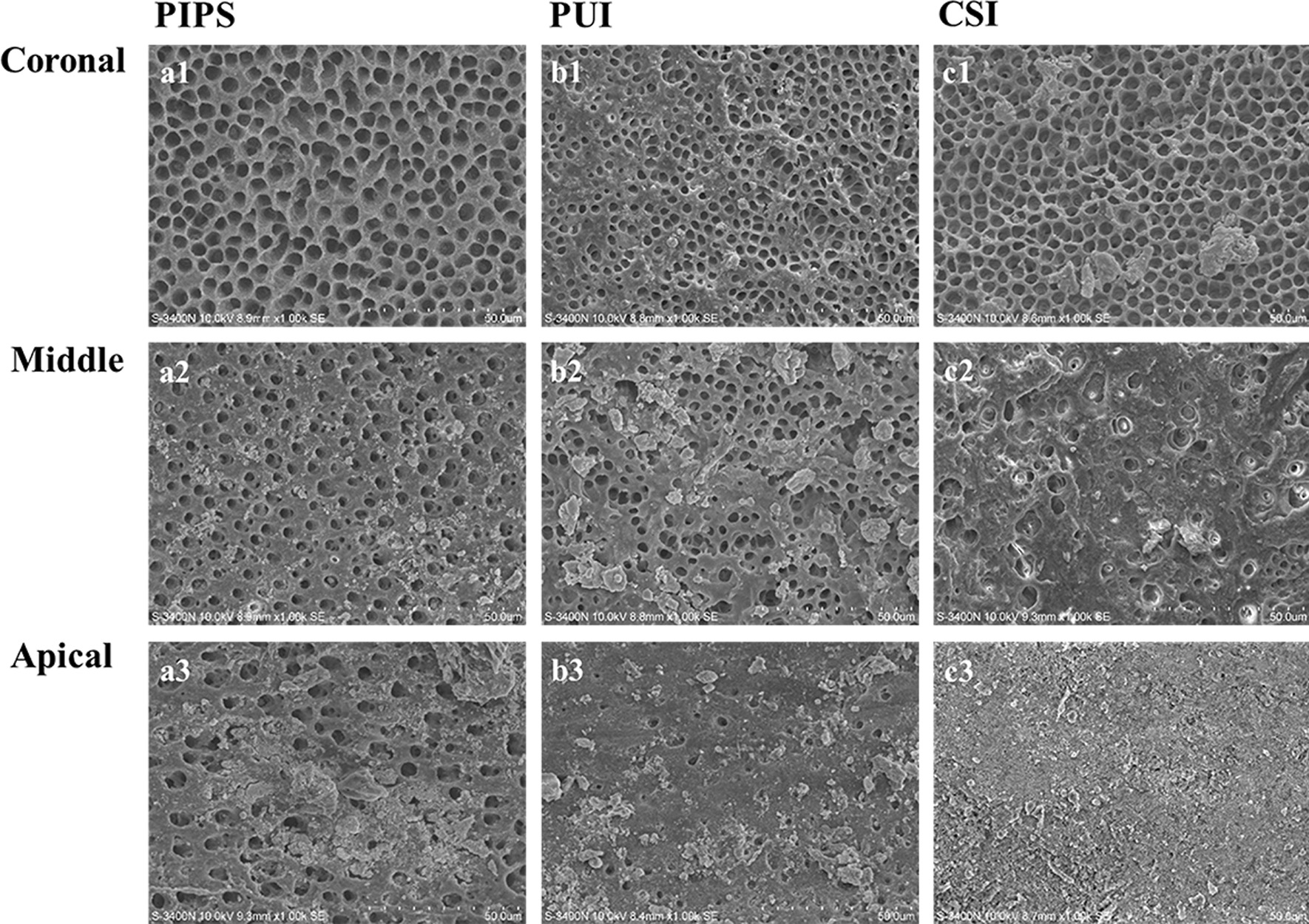
Scanning electron microscopy (SEM) photomicrographs of the remaining filling materials in the coronal, middle, and apical thirds of the root canal (×1000): after photon-induced photoacoustic streaming (PIPS) (**a1** coronal third; **a2** middle third; **a3** apical third); after passive ultrasonic rinsing (PUI) (**b1** coronal third; **b2** middle third; **b3** apical third); after classic syringe-based irrigation (CSI) (**c1** coronal third; **c2** middle third; **c3**, apical third)

Representative SEM images were taken from all canal thirds of the samples (Fig. 2). In the apical third, the CSI group showed a thick smear layer and filling debris covering nearly all of the canal walls. The PUI group showed some dentinal tubules open and others covered by a thin smear layer and filling debris; less than 50% of tubules were visibly exposed in a limited area. The PIPS group showed small amounts of smear layer and filling debris and 50 ~ 75% of dentinal tubules opened. In the middle third, the CSI group showed most of the canal walls covered with smear layer and filling debris, the PUI group showed small amounts of smear layer and filling debris and some dentinal tubules opened, while the PIPS group showed small amounts of smear layer and filling debris and > 75% of tubules exposed. In the coronal third, the CSI and PUI groups showed small amounts of smear layer and filling debris and > 75% of dentinal tubules opened, and the PIPS group showed most of the tubules opened and no smear layer and filling debris.

As shown in Table 3, in the coronal third, the scores of the three groups were not significantly different (*p* > 0.05). In the middle and apical thirds, the scores were significantly lower in the PIPS group than in the PUI (*p* < 0.05) and CSI groups (*p* < 0.05), and were also significantly lower in the PUI group than in the CSI group (*p* < 0.05). Within each group, the scores of the three thirds were significantly different, with the apical third less than the coronal third (*p* < 0.05). The two examiners had a high intraclass correlation coefficient (ICC) in SEM image evaluation (ICC value = 0.843).

**Table 3:** Median, maximum and minimum SEM scores for cleanliness of the canal walls in each third after each final irrigation-activation technique

Groups	CSI	PUI	PIPS
	Median (Minimum-Maximum)		
Coronal	1 (0–2)^a1^	1 (0–1)^a1^	0 (0–2)^a1^
Middle	2 (0–2)^a1^	1 (0–2)^a2^	1 (0–2)^ab3^
Apical	3 (2–3)^c1^	2 (1–3)^b2^	1 (0–3)^b3^

ab Ranking: statistically significant differences among the coronal, middle, and apical thirds within each group (*p* < 0.05)

123 Ranking: statistically significant difference among groups (*p* < 0.05)

## Discussion

To satisfy periapical healing after root canal retreatment, the previous filling materials should be adequately removed, and the patency and the WL should be established during retreatment [31]. iRoot SP sealer based on a calcium silicate composition has the potential to adhere chemically to dentin [32]. Along the material–dentine interface, calcium silicate–based material could form a tag-like structure that was composed of either Ca- and P-rich crystalline deposits or the material itself, which may lead to good marginal sealing ability and dentine bonding ability [33]. These may be the reasons for why the BC sealer has always been found to leave significantly more residual filling materials compared to AH Plus or AH-26 in the root canals obturated by continuous wave compaction technique, single-cone technique or lateral compaction technique [17, 34]. Oltra et al. used ProFile files to remove the gutta-percha and the BC sealer in the root canal and found that there was still some sealer remaining in the canal, especially in the apical third [17]. Uzunoglu et al. found that after ProTaper Universal Retreatment instrumentation, residual iRoot SP in the apical and middle thirds of the canals was similar to or higher than the coronal thirds [34]. Therefore, the retreatability of iRoot SP is still a challenge and a more effective technique is needed.

In this study, PUI and PIPS were used as additional methods to remove the residual gutta percha and iRoot SP sealer after mechanical retreatment. Our results demonstrated that none of the additional techniques in this study completely removed the residual iRoot SP and gutta-percha. However, the additional use of PIPS after mechanical retreatment resulted in a significant improvement in removing the iRoot SP and gutta-percha. Similarly, previous studies have shown that PIPS used after NiTi retreatment instruments could improve the removal of the AH plus sealer [23, 25]. Our study also found that PIPS and PUI were superior to CSI in removing residual material from the apical third of the canal. This finding indicated that PIPS and PUI performed effectively as additional techniques after the use of NiTi instrumentation in endodontic retreatment to remove the residual material in the apical canal. SEM results showed that the scores of the three groups were not significantly different in the coronal third, while in the middle and apical thirds, the cleanness levels of the three groups were significantly different, with the PIPS group showed the best cleanliness, and the CSI group showed the worst. All of these findings indicated that PIPS could greatly reduce the smear layer and filling debris on the canal wall and open the dental tubules. Thus the null hypothesis that there was no difference among the three retreatment techniques was rejected.

The better effect of PUI and PIPS on removal of the residual material in the root canals may be related to their mechanism. PUI causes an acoustic flow by producing a rapid circular and swirling motion in the irrigants, and induces a cavitation effect around the ultrasonic file [35]. Similarly, the laser used for PIPS irradiates the irrigant; the subsequent vaporization of the irrigant results in the formation of vapor bubbles, which expand and implode with cavitation effects. The irrigant could rush into the bubble from the back, making the imploding bubble become shaped like a sickle [36]. Moreover, a previous study showed that compared with PUI, PIPS could greatly promote the penetration of solution into the dentinal tubules especially in the apical part of the canal. Due to the anatomical conditions and accessibility in the apical area, PIPS might be advantageous in the removal of filling materials in the apical third of the canal, as it does not depend on the insertion depth of a file or probe, such as PUI or CSI used in this study [20]. These factors may be responsible for our finding that in the middle and apical thirds of the canals, cleanness was greater in the PIPS group than in the PUI group.

PIPS for root canal irrigation most commonly used an Er:YAG laser. In some in vitro studies, different parameters of the Er:YAG laser were used for PIPS. Jiang, et al. used an Er:YAG laser with 50 mJ, 20 Hz, 1 W to remove AH Plus sealer and gutta-percha [25]. Keles, et al. used an Er:YAG with 45 mJ, 20 Hz, 0.9 W to remove the filling debris from the canal [23]. Since the energy of an Er:YAG laser is greatly absorbed by water, PIPS needs only a low energy to achieve a better activation-irrigation effect. In the clinic, a small energy of 20 mJ and a short pulse duration are recommended for the Er:YAG laser [37], and the water/gas function needs to be turned off. With a higher power setting (more than 20 mJ), splashing of irrigant outside the tooth may occur, which may cause loss of irrigant and ineffective irrigation. Moreover, the irrigation needs to remain in the root canal to avoid direct laser irradiation of the dentin wall, which may cause thermal damage. The results of this study showed that an Er:YAG laser with 20 mJ, 15 Hz, and 0.3 W can effectively clean the tricalcium silicate-based filling materials, consistent with a previous study that found that an Er:YAG laser with these settings could effectively remove AH Plus, EndoSequence BC and MTA Fillapex sealers [24].

NaOCl and EDTA are the solutions most commonly used as irrigants for root canal irrigation of PIPS [38, 39]. NaOCl can dissolve organic tissues and disinfect the root canal. The generally used concentration is 0.5–5.25%. However, high-concentration NaOCl overflowing from the apical foramen may cause severe complications [40]. Our results showed that PIPS with 2.5% NaOCl and 17 %EDTA effectively reduced the smear layer and filling debris on the canal wall surface and opened the dentin tubules, thus facilitating irrigant penetration. Therefore, low-concentration NaOCl could also obtain an ideal effect in endodontic retreatment, which may improve safety. Based on the above, activation of 2.5% NaOCl and 17% EDTA with PIPS could be used as an additional technique to remove residual tricalcium silicate-based sealers and gutta-percha after the use of NiTi instruments in endodontic retreatment.

## Conclusions

None of the additional techniques in this study completely removed the residual iRoot SP and gutta-percha. Compared to PUI and CSI, activation of 2.5% NaOCl and 17% EDTA with PIPS greatly improved the removal of the residual iRoot SP and gutta-percha following NiTi mechanical retreatment.

## Data Availability

All data are available from the corresponding author upon reasonable request.

## Abbreviations

NiTi: Nickel–titanium
PTUR: ProTaper Universal retreatment
PUI: Passive ultrasonic irrigation
PIPS: Photon-initiated photoacoustic streaming
Er: YAG:erbium:yttrium-aluminum-garnet
CSI: Classic syringe-based irrigation
micro-CT: Microcomputed tomography
SEM: Scanning electron microscopy
WL: Working length
PTN: ProTaper Next
NaOCl: Sodium hypochlorite
EDTA: Ethylenediaminetetraacetic acid
ICC: Intraclass correlation coefficient
